# A study of best positive predictors for sustained virologic response to interferon alpha plus ribavirin therapy in naive chronic hepatitis C patients

**DOI:** 10.1186/1471-230X-9-5

**Published:** 2009-01-20

**Authors:** Muhammad Idrees, Sheikh Riazuddin

**Affiliations:** 1 Division of Molecular Virology & Molecular Diagnostics, National Centre of Excellence in Molecular Biology, University of the Punjab, Lahore 53700, Pakistan

## Abstract

**Background:**

The aim of this study was to determine the rate of sustained virological response (SVR) and various factors associated with response rates in chronic hepatitis C infected patients treated with interferon alpha and ribavirin combination therapy.

**Methods:**

A retrospective review of patients data collected at this Centre from 2001 to 2007 was performed. Out of 731 consecutive patients 400 patients that fulfilled the study criteria were evaluated and subsequently treated with a combination of interferon alpha 2b (3 MU subcutaneously three injections weekly) and ribavirin (800–1200 mg orally daily). Treatment were administered for either 24 weeks or 48 weeks and patients were followed for an additional 6 months thereafter. End of the treatment response (ETR), SVR and side effects were recorded.

**Results:**

Out of 400 patients, 394 completed the entire treatment course and six patients discontinued treatment at month 2. Over 67% responded at the end of treatment and 16% suffered relapse. Among all treated patients, 47.6% males and 56.7% females had sustained viral response with a total combined sustained viral response rate of 50.5%. Rapid response was seen in 46.5% patients. In a multivariate logistic regression analysis, slow virological responders (adjusted OR 2.6 [95% CI 1.9–3.7]), HCV genotype 1&4 (adjusted OR 2.4 [95% CI 1.7–3.5]), pre-treatment viral load > 0.2 MIU/mL (adjusted OR 2.2 [95% CI 1.8–4.2]), Panjabi ethnic group (adjusted OR 1.6 [95% CI 1.0–3.2]) and Age > 40 years (adjusted OR 1.5 [95% CI 0.9–2.4]) were independent risk factors for non response. Side effects were usual and tolerable and only 1.5% discontinued the treatment.

**Conclusion:**

The best positive predictor for SVR in this country are: rapid virologic response, HCV genotype 2 & 3, age < 40 years, ethnic race Pashtoons and pre-treatment viral load < 0.2 million IU/mL.

## Background

Hepatitis C virus (HCV) is one of the most important *Flaviviridae *infections in humans and is the second most common cause of viral hepatitis [1]. Currently, almost 8–10% of the Pakistani population (MI., SR. unpublished data), 2% of the United States of America (USA) population, and an estimated 170 million people worldwide are HCV carriers [2]. Chronic HCV infection frequently results in liver cirrhosis and is associated with an elevated risk of developing hepatocellular carcinoma [3]. Although symptoms may be mild for decades, 20% of persistently infected individuals may eventually develop serious liver disease including cirrhosis and liver cancer [2]. The only effective treatment is based on interferon alpha (IFN-α). Treatment with either IFN-α alone or in combination with ribavirin leads to a sustained virological response (SVR) in 20% to 56% of patients with chronic hepatitis C [4,5]. The combination of interferon and ribavirin is the preferred treatment and achieves a better response than interferon or ribavirin alone [6]. However, nonresponse to this therapy remains common and is associated with several factors such as HCV genotype, duration of a person's HCV infection and HCV viral load in addition to host factors such as sex, HLA type and cytokine polymorphisms [7,8]. Patient age, grade of liver inflammation and ethnicity have also been shown to influence response to therapy [9,10]. The strongest predictors for a SVR to treatment is the HCV genotype, with HCV genotype 1 (HCV-1) being the least sensitive to IFN-α based therapy [11,12].

Several studies are available on the response rates to combinatorial IFN-α/ribavirin treatment of hepatitis C in Pakistan [13,14], however, these do not describe positive and negative predictors for the SVR rates. The aim of this study was to determine the efficacy and safety of a 24 weeks or 48 weeks treatment with IFN-α plus ribavirin in patients with chronic hepatitis C genotypes none-1 and 1 respectively and to identify factors that impaired response to antiviral therapy. We focused our study on naïve patients that had not previously received antiviral treatment and who presented with HCV genotypes non-1 and 1.

## Methods

### Patients

A retrospective analysis was performed on data collected from 731 patients with chronic HCV infection screened between 2001 and 2007 at multiple clinics throughout Pakistan. Of the 731 consecutive screened patients, 400 patients (280 male, 120 female, mean age 38 years [range, 16–70 ± SD] years) were chronically infected with various HCV subtypes and had not received antiviral treatments previously. These 400 patients fulfilled the study criteria and were enrolled for antiviral therapy. A total of 331 patients were excluded as either they were unwilling to participate (n = 119) or failed to meet inclusion criteria for the study (n = 212). Patients were enrolled from provinces of Punjab (Panjabi; 299), North West Frontier Province (Pashtoons; 78), Sindh (Sindhi; 11) and Balochistan (Balochi; 12). Probable transmission risk factors were previous major/minor surgery (20%), transfusion of blood or blood products (2%), dental surgery (10%), or sporadic (60%). The estimated duration of infection varied from 6 months to 20 years.

### Diagnosis, inclusion and exclusion criteria

The diagnosis of chronic hepatitis C was based on elevated serum transaminase levels for at least 6 months and the consistent detection of serum HCV RNA. Anti-HCV antibodies (3^rd ^generation ELISA) were present in each patient. Prior to the start of the treatment, patients complete blood count (CBC) were measured and patients were required to have haemoglobin levels above 13 g/dL in males and 12 g/dL in females and platelet counts above 80,000. Patients were also required to have persistently elevated alanine aminotransferase (ALT), white blood cell count greater than 3.0/mm^3^, normal serum bilirubin, albumin, creatinine and thyroxin levels. Furthermore the patients were required to be negative for hepatitis B surface antigen, and not suffering from decompensate liver disease, autoimmune disorders, or a history of depression and/or cardiac diseases. Patients that were under 18 years or above 70 years of age and pregnant females were also excluded from the studies.

### Measurement of HCV RNA and HCV genotyping

Quantitative detection of HCV RNA in patient sera during pre-treatment, treatment and post-treatment were performed using real-time PCR (SmartCycler II, Cepheid, USA) with an internal RNA standard derived from the 5' UTR. Genotyping of HCV was performed in pre-treatment sera as described previously [15]. All measures suggested by Kwok and Higuchi [16] to prevent sample contamination were strictly adhered to.

### Treatment

All patients with HCV 2a-b, 3a-d, 5a, 6a or with mixed infection of these genotypes received 3 subcutaneous injections per week of 3 MU recombinant IFN-α plus ribavirin (10 mg/day/kg body weight) for a total of 24 weeks. All patients with HCV genotypes 1a-c, 4 or those with mixed infections of these genotypes received 3 subcutaneous injection per week of 3 MU recombinant IFN-α plus ribavirin administered orally (1,000 mg/day for patients ≤ 75 kg body weight or 1,200 mg/day for patients > 75 kg body weight) for 48 weeks. Informed consent (printed in local language) was obtained from each patient and the Ethics Committee of the Centre approved this study.

### Patients monitoring

All patients that fulfilled the study criteria were monitored for HCV RNA and ALT during and after treatment. Efficacy of treatment was assessed with normalization of ALT and absence of serum HCV RNA measured at week 4, 8, 12 and 24 and being undetectable at the end of the treatment at 24 or 48 weeks which constituted end of treatment response and at the end of follow up at 12 or 18 months which constituted the sustained viral response for patients with genotypes non-1 and 1 respectively. Adverse effects were monitored during each follow up visit. The study was initiated in May 2001 and all the follow-ups were completed by June 2007. A number of study end-points were defined, based upon the copy number of HCV RNA in patient sera. End of treatment response (ETR) was defined as undetectable HCV RNA (< 100 copies/mL) at the end of treatment. Sustained virologic response (SVR) was undetectable HCV RNA 6 months after the end of treatment and non responders were those who were with detectable HCV RNA at the end of treatment. Patient relapse was defined as those whose HCV RNA was undetectable at the end of treatment but reappeared within 6 months of cessation of treatment. Rapid virologic response (RVR) was defined as undetectable HCV RNA following an initial 4 weeks of treatment.

### Statistical analysis of the generated data

Clinical and biochemical characteristics of patients are expressed as a mean or median, with a standard deviation as appropriate. Summary statistics were calculated using SPSS (v10.0 SPSS Inc.). The results for all variables were given in the form of rates (%.). Chi-square and Fishers Exact test was used to measure association amongst categorical variables. *P*-values less than 0.05 were considered significant. Multivariate logistic regression analysis was performed to identify factors that were associated with non response to therapy. Variables associated with relapse in univariate analysis (P < 0.1) were entered into the model. Potential confounders were tested before selecting the final model by entering non-significant variables into the selected multivariate model and determined their effect on the odds ratios of the independent variables.

## Results

### Study enrolments and disposition of patients

**Figure 1:**
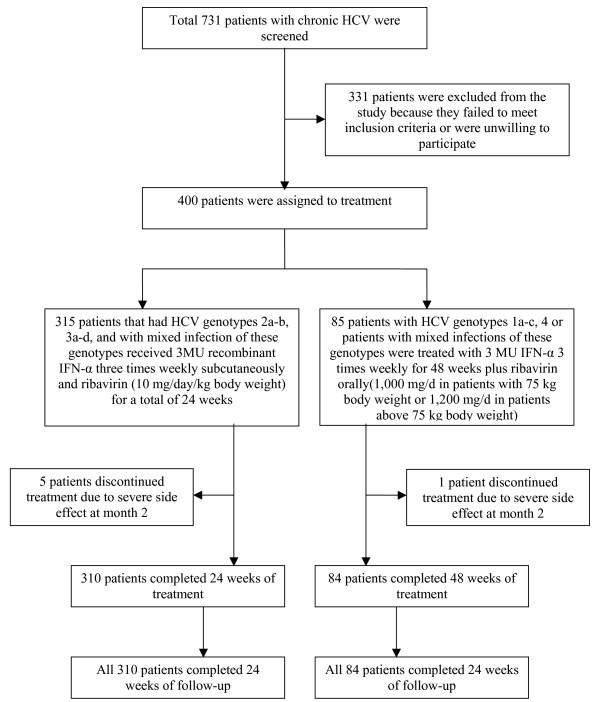
**Study enrollment and disposition of patients**.

Four hundreds patients with chronic HCV who fulfilled the study criteria were enrolled for this study from an initial pool of 731 patients (Figure 1). Of these 400 patients, 280 were males and 120 females. Six patients discontinued treatment due to severe side effects experienced within the second month of treatment. Four patients displayed a rapid virological response with negative PCR at the time of discontinuation of treatment. Two hundred and seventy six males and 118 females, making a total of 394 patients completed the entire course of treatment. A total of 57 patients had liver biopsies out of which 16 had liver cirrhosis and rest of the 41 had stage 1–3 of fibrosis.

The demographic characteristics of the patients treated are shown in Table 1. A total of 331 patients were excluded from the study either they were unwilling to participate in the study (n = 119) or failed to meet inclusion criteria of the study (n = 212) such as low haemoglobin level (below 13 g/dL in men and 12 g/dL in women), and/or low platelet counts (below 80,000) and/or white blood cell count less than 3.0/mm^3^, abnormal serum bilirubin, albumin, creatinine and thyroxin levels. Six patients were also positive for hepatitis B surface antigen, four were with decompensating liver disease, five were with autoimmune disorders and 23 had history of depression and cardiac diseases. Pregnant women (n = 9), patients < 18 years or > 70 years (n = 7) and with persistently normal ALT levels (n = 61) were also excluded from the study.

**Table 1:** Demographic characteristics of treated patients (N = 400).

Characteristics	Genotype 1 (N = 70)	Genotype 2 (N = 33)	Genotype 3 (N = 260)	Other Genotypes (N = 6) *	Mixed Genotypes (N = 31)^†^
**Sex-No. (%)**					
Male	47 (67.1)	22 (66.7)	185 (71.2)	6 [100]	20 (64.5)
Female	23 (32.9)	11 (33.3)	75 (28.8)	0 [0]	11 (35.5)

**Age range-years**					
Mean age (Y)	25–70	21–68	18–55	20–57	21–60
± SD^‡^	50 ± 9.5	48 ± 9.0	45 ± 10.2	49 ± 9.8	46 ± 8.4

**Ethnic group-No. (%)**					
Panjabi	50 (71.4)	21 (63.6)	203 (78.1)	4 (66.7)	21 (67.7)
Pashtoons	15 (21.4)	10 (30.3)	41 (15.8)	2 (33.3)	10 (32.3)
Balochi	3 (4.2)	1 (3.0)	7 (2.7)	0	0
Sindhi	2 (2.9)	1 (3.0)	9 (3.4)	0	0

**Pre-treatment HCV RNA level**					
< 200,000 IU/mL	22 (31.4%)	11 (33.3)	71 (27.3)	1 (16.7)	4 (12.9)
> 200,000 IU/mL	48 (68.6)	22 (66.7)	189 (72.7)	5 (83.3)	27 (87.1)

**Cirrhosis-No (%)**					
Present	3 (4.3)	2 (6.1)	9 (3.5)	0 [0]	2 (6.5)
Absent/unknown	67 (95.7)	31 (93.9)	251 (96.5)	6 [100]	29 (93.5)

*Of the patients with other genotypes, 4 had HCV genotype 4 and one each was with genotype 5a and 6a.

^†^Out of 31 patients with mixed genotypes, 13 were with genotypes 3a+3b; 4 with 2a+3b; 3 each with 2a+3a & 1a+1b, 2 each with 1a+3a & 3a+4; 1 each with 1a+3c, 1b+2a, 1c+3b & 1c+3c.

^‡^SD, standard deviation.

### Virologic response

Out of 400 patients, 268 patients (67%) showed end of treatment response (ETR) and 202 (50.5%) patients achieved a sustained virological response (SVR). Sixty six (16.5%) patients were HCV-RNA negative at the end of treatment but relapsed thereafter. One hundred and thirty two (33%) patients were virologic nonresponders showing detectable HCV RNA at the end of treatment. Rapid virological response was seen in 186 (46.5%) patients. Out of sixteen patients who had cirrhosis on the liver biopsy, 10 were males and 6 were females. Half of these patients had end of treatment response but all were relapsed giving no sustained viral response in these patients.

### Virologic response in different ethnic groups

An ethnic difference was seen in both ETR and SVR rates. In ethnic groups Pashtoon, Panjabi, Sindhi and Balochi the rates of SVR were 69.2%, 45.5%, 45.5% and 50% respectively. The rates of SVR are significantly higher in ethnic group Pashtoon compared with Panjabi, Sindhi and Balochi patients (P = 0.026).

### Predictors of sustained virologic response

Several factors were found to be predictors of SVR in this study. These were HCV genotype, pre-treatment HCV RNA levels, age, presence or absence of cirrhosis, rapid virologic or delayed virologic responses and ethnicity. Tables 2 summarize the ETR, SVR or no response (NR) results in patients with different HCV genotypes. It is clear from the data of the present study that the rates of ETR and SVR are different among patients with different genotypes. The highest SVR was seen in patients with HCV genotype 2 (69.7%) followed by genotype 3 having patients (57.3%) and lowest SVR in genotype 1 infected patients (24.3%). Table 3 shows rates of ETR, SVR and NR in patients with different age groups. Rates of ETR and SVR are very high in patient ≤ 20 years (66.7%) and very low (0%–27%) in age group ≥ 50 years. All the variables associated with SVR in chronic HCV patients studied in the course of the present study are shown in Table 4. The SVR was found significantly higher in patients below 40 years of age compared to those above 40 years (P = 0.022), having non-1 genotype than patients with genotype 1 & 4 (P < 0.001), patients with pre-treatment viral load of < 0.2 million IU/mL compared with pre-treatment viral load of > 0.2 million IU/mL blood (P < 0.001), rapid virologic response (RVR) achiever than non-RVR achiever (P = 0.04), absence of cirrhosis compared to its presence (P = 0.002) and in ethnic groups Pashtoon compared with Panjabi patients (P = 0.026). Pre-treatment ALT levels had no predictive role in treatment response rate. No significant difference was seen for ETR and SVR in male and female patients.

**Table 2:** Rates of NR*, ETR^† ^and SVR^‡ ^in patients infected by different HCV genotypes to interferon plus ribavirin therapy (N = 400).

HCV GENOTYPE	TREATED PATIENTS	NR*	ETR^†^	SVR^‡^
1 (a, b, c)	70	42 (60%)	28 (40%)	17 (24.3%)

2 (a, b)	33	5 (15.2%)	28 (84.8%)	23 (69.7%)

3 (a, b, c, d)	260	65 (25%)	195 (75%)	149 (57.3%)

Others (4, 5a, 6a)	6	2 (33.3)	4 (66.7%)	3 (50%)

Mixed^ψ^	31	18 (58.1%)	13 (41.9%)	10 (32.3%)

**Total**	**400**	**132 (33%)**	**268 (67%)**	**202 (50.5%)**

*NR, Non-responders, defined as detectable HCV RNA at the end of anti-viral treatment.

^†^ETR, End of treatment response, defined as undetectable HCV RNA at the end of treatment.

^‡^SVR-Sustained virologic response, defined as undetectable HCV RNA 24 weeks after end of anti-viral treatment.

^ψ^Of the 31 patients with mixed genotypes, 13 were with genotypes 3a+3b; 4 with 2a+3b; 3 each with 2a+3a & 1a+1b, 2 each with 1a+3a & 3a+4; 1 each with 1a+3c, 1b+2a, 1c+3b & 1c+3c.

**Table 3:** Rates of NR*, ETR^† ^and SVR^‡ ^in HCV infected patients of different Age groups to interferon plus ribavirin therapy (N = 400).

**S. No**.	AGE GROUP	TOTAL PATIENTS	NR (%)	ETR (%)	SVR (%)
1	≤ 20 Years	9	1 (11.1%)	8 (88.9%)	6 (66.7%)

2	21–30 Years	91	27 (29.7%)	64 (70.3%)	50 (54.9%)

3	31–40 Years	154	48 (31.2%)	106 (68.8%)	88 (57.1%)

4	41–50 Years	107	40 (37.4%)	67 (62.6%)	48 (44.9%)

5	51–60 Years	37	14 (37.8%)	23 (62.2%)	10 (27.1%)

6	> 60 Years	2	2 (100%)	0	0

Total		400	132 (33%)	268 (67%)	202 (50.5%)

*NR, Non-responders, defined as detectable HCV RNA at the end of anti-viral treatment.

^†^ETR, End of treatment response, defined as undetectable HCV RNA at the end of treatment.

^‡^SVR-Sustained virologic response, defined as undetectable HCV RNA 24 weeks after end of anti-viral treatment.

**Table 4:** Variables associated with rates of SVR^‡ ^in chronic HCV patients treated with interferon plus ribavirin standard therapy (N = 400).

Variable	Total No. of treated patients (n = 400)	Achieved SVR (n = 202)	SVR rate, %	P-value
**Age, years**				
≤ 40	251	144	57.4	0.022
> 40	149	58	38.9	

**Sex**				
Female	120	68	56.7	NS
Male	280	134	47.8	

**Ethnic group**				
Pushtoon	78	54	69.2	0.026
Punjabi	299	136	45.5	
Sindhi	11	5	45.5	
Baloch	12	6	50.0	

**Pre-treatment viral load**				
< 0.2 MIU/mL	291	173	59.5	0.001
> 0.2 MIU/mL	109	29	26.6	

**Rapid virological response**				
Achieved	186	139	74.7	0.04
Not achieved	214	63	29.4	

**HCV genotype**				
2, 3 or mixed with 2&3	315	181	57.5	0.005
1, 4, 5, 6 or mixed	85	21	24.7	

**Cirrhosis^†^**				
Present	16	0	0	0.021
Absent	41	23	56.09	

**Pre-treatment ALT**				
Moderately high	239	121	50.6	NS
Very high	161	81	50.3	

^‡^SVR-Sustained virologic response, defined as undetectable HCV RNA 24 weeks after end of anti-viral treatment.

^†^The number of participants does not add up to400 for one variable due to it being not applicable.

Multivariate logistic regression analysis indicated that non achiever of RVR (adjusted OR 2.6 [95% CI 1.9–3.7]), HCV genotype 1&4 (adjusted OR 2.4 [95% CI 1.7–3.5]), pre-treatment viral load > 0.2 MIU/mL (adjusted OR 2.2 [95% CI 1.8–4.2]), Panjabi ethnic group (adjusted OR 1.6 [95% CI 1.0–3.2]) and Age > 40 years (adjusted OR 1.5 [95% CI 0.9–2.4]) were independent risk factors for low rates of SVR (Table 5).

**Table 5:** Multivariate logistic regression analysis of variables associated with decrease sustained virological response rates to alpha interferon plus ribavirin treatment in chronic HCV patients (N = 400).

Variable	SVR^‡^/Total No. of treated patients	Adjusted OR*	95% CI^ψ^
**Age, years**			
> 40	58/149	1.5	0.9–2.4
≤ 40	144/251		

**Ethnic group**			
East (Punjab)	136/299	1.6	1.0–3.2
North-west (NWFP)	54/78		

**Pre-treatment viral load**			
> 0.2 MIU/mL	29/109	2.2	1.8–4.2
< 0.2 MIU/mL	173/291		

**Rapid virological response**			
Achieved	139/186	2.6	1.9–3.7
Not achieved	63/214		

**HCV genotype**			
1, 4, 5, 6 or mixed with these genotypes	21/85	2.4	1.7–3.5
2, 3 or mixed with these genotypes	181/315		

**Cirrhosis**			
Present	0/16	--	CI cannot be
Absent	23/41		calculated

*OR, Odd ratio

^ψ^CI, Confidence interval

^‡^SVR-Sustained virologic response, defined as undetectable HCV RNA 24 weeks after end of anti-viral treatment.

^†^The number of participants does not add up to 400 for one variable due to it being not applicable.

### Safety

All patients tolerated the treatment well except for six patients who discontinued due to severe side effects. Dosage reduction of ribavirin was made in 10 patients due to drop in haemoglobin level below 10 g/dL and the interferon units were reduced in 5 patients due to drop in leukocytes counts below 2,000 and platelet count below 50,000. All these patients completed the treatment with adjusted dosage. The side effects were mostly influenza-like syndrome, which occurred in more than 80% patients. Gastrointestinal, psychiatric, dermatological symptoms and other side effects were tolerated. The side effects observed in patients during therapy are detailed in Table 6.

**Table 6:** Side effects observed during anti-viral therapy in HCV infected patients (N = 400)

Side effect observed	Percent patients
Influenza like symptoms	90%
Fever	85%
Fatigue	68%
Headache	40%
Myalgias/Arthralgias	86%
Anorexia	25%
Nausea/Vomiting	21%
Abdominal pains	20%
Insomnia	18%
Anxiety	5%
Irritability	5%
Psychosis	5%
Suicide attempt	0.5%
Redness at injection site	8%
Pruritis	75%
Dry skin	50%
Anaemia	45%
Leucopoenia	2%
Thrombocytopoenia	1%
Laryngitis	1%

## Discussion

Our study of patients infected with HCV shows that the overall end of treatment response rates to interferon plus ribavirin therapy is very high (67%) in this country. In a considerable proportion of patients (16%) however, virologic relapse occurred after the completion of therapy in the follow up period. This relapse rate is very high as compared to that reported by Khokhar and Co-workers [17] in Pakistani patients (only 4% relapse rate) but is very low compared to that reported by Sarrazin et al., [18] that was 43%. Similarly, John et al., [19] have reported a response rate of 53% to combination therapy for 6 months with sustained virological response of 31%. The low response rate reported by Sarrazin et al., and John et al., may be due to the fact that genotype 1b was predominant in their study which is associated with severe clinical course of the disease and a decreased response to treatment. Conversely, in Pakistan the predominant genotype is 3a, which has a slower progression and a better response to treatment [20].

A number of viral and host factors have been identified during the course of the present study that may help to predict response rates of current antiviral therapy. Our data suggest that the patients slow/rapid virologic response rate, their pre-treatment HCV viral load, age, stage of fibrosis, gender, ethnicity and HCV genotypes are factors that may play an important role in determining response rates to interferon plus ribavirin treatment. The most important of these factors appears to be the achievement of undetectable level of HCV-RNA in serum by week 4, the HCV genotype and pre-treatment viral load. This data suggest that patients with a rapid virologic response (RVR) had a significantly higher sustained virologic response (SVR) rate (P < 0.05); in more than 85% of cases RVR occurred with SVR. These findings support a recent study that suggests high rates of SVR in patients shown RVR within 16 weeks and 24 weeks of therapy [21]. As expected, it was also observed in the present study that rate of SVR differed among patients with different HCV genotypes, with higher SVR observed with HCV genotypes 2 and 3. This finding in the Pakistani population confirms previously reported work that shows a better response to IFN-alpha treatment in patients with HCV genotypes 2 and 3 [8,22,23] from other parts of the World. An interesting finding of the present study is that the SVR rate is significantly higher in patients with a pre-treatment viral load of less than 200,000 IU per mL than patients with pre-treatment viral load of greater than 200,000 IU/mL. Several other recent studies have shown an inverse relationship between sustained virologic response rates and pre-treatment viral load [21,24].

Perhaps the most interesting and important finding of the present study is the observation that response rates are significantly higher (P = 0.026) in ethnic Pashtoon as compared to Panjabi. Race has also been shown to influence response to therapy by other researchers [9,10]. This suggests that Panjabi race is an independent risk factor for the decreased rate of response to treatment of HCV infection. These data need to be conformed by further studies with larger numbers of Pashtoon patients. The baseline characteristics of Pashtoon and Panjabi patients were similar however we were unable to assess differences in body mass index and incidence of diabetes mellitus that may play a role in interferon non-responsiveness. Similar rates of SVR have been reported in Indian Panjabi 53% [25] compared to 45.5% seen in the present study. The 7.5% greater response rate seen in Indian Panjabi race may be due to the administration of pegylated interferon and ribavirin for treatment. Our study further shows that younger patients have higher response rates to antiviral therapy than older patients (P < 0.001) in agreement with previous studies [21-23]. Another important finding of the present study is that patients with cirrhosis had 0% SVR rates even though 85% patients with cirrhosis enrolled in this study had ETR. This observed ETR is much better than previously reported response but unfortunately all of our patients with cirrhosis were relapsed soon after treatment was discontinued.

All patients enrolled in this study tolerated the treatment well and the side effects were in line with that previously reported [26]. Six of our patients did develop psychosis and had to discontinue treatment after 2 months of treatment, relapsing soon thereafter. Though side effects of the combination regimen are additive not synergistic, they generally do not limit the safe use of this therapy. Appropriate recognition and management of side effects will both improve therapeutic response and avoid unnecessary morbidity and mortality [27].

## Conclusion

In conclusion our study shows that twenty-four weeks combination treatment with interferon-α2b and ribavirin in patients with genotypes 2 and 3 may be as useful as PEG-interferon plus ribavirin for sustained virological response. Patients with RVR, low pre-treatment viral load, HCV genotypes 2 & 3, age < 40 years and ethnic group Pashtoon appear to have the highest probability of ETR and SVR.

## Competing interests

None of the authors who participated in this study have commercial or other associations that might creat conflict of interest.

## Authors' contributions

MI and SR conceived the study participated in its design and execution, and wrote the manuscript. MI collected clinical data, performed the statistical analysis and carried out all the molecular, quantitative and genotyping assays. All authors have read and approved the final manuscript.

## Pre-publication history

The pre-publication history for this paper can be accessed here:

http://www.biomedcentral.com/1471-230X/9/5/prepub
